# Long-term control in chronic rhinosinusitis after endoscopic sinus surgery

**DOI:** 10.1186/2045-7022-3-S2-P44

**Published:** 2013-07-16

**Authors:** Julie van der Veen, Marieke Timmermans, Mark Jorissen, Peter Hellings

**Affiliations:** 1 University Hospitals Leuven, Department Otorhinolaryngology-Head & Neck Surgery, Leuven, Belgium

## Background

The concept of control of chronic rhinosinusitis (CRS) has only recently been introduced by the EPOS expert panel following the concept of control in allergic rhinitis and asthma. The aim of this study was to examine the degree of control of CRS at a mean period of 3-5 years after endoscopic sinus surgery (ESS) and to correlate the new criteria of control of CRS to VAS (visual analogue scores) for sinonasal symptoms and the SNOT-22 questionnaire. Another aim was to find out if patients changed from controlled to partly controlled or uncontrolled following nasal endoscopy.

## Methods

All adult patients who had a bilateral ESS procedure for bilateral inflammatory sinonasal disease at the University Hospitals Leuven between January 2008 and December 2010 were contacted by classic mail. They were asked to complete a survey including sinonasal symptoms according to EPOS criteria, VAS scores, SNOT-22 and SF-36 questionnaires and general questions regarding asthma, smoking habits, allergies and current medication intake. They were invited to the out-patient clinic for nasal endoscopy.

## Results

The response rate was 54.5% (n = 297). 19.8% were well controlled, 39.3% partly and 40.9% uncontrolled. There was no significant difference between the CRSwNP and CRSsNP groups. These results only depended on the self-reported symptoms, without taking into consideration endoscopic examination. Sixty patients underwent endoscopic examination of which 10% were controlled, 30% partly and 60% uncontrolled. The results of the examination showed that 18 patients had signs of recurrence (30%). In 77.8%, the endoscopic findings did not change the degree of control as defined by the questionnaire. 16.7% changed from partly to uncontrolled and 5.6% from controlled to partly controlled. The average global VAS scores of the controlled, partly and uncontrolled groups were 0.8, 2.7 and 5.7 respectively. The average SNOT-22 scores were 9.7, 22.2 and 44.8 respectively.

## Conclusion

The percentage of partly and uncontrolled CRS greatly overshadows the 19.8% of well controlled CRS. In 77.8% of cases, endoscopic investigation did not change the degree of self-reported control. The sensitivity of the EPOS questionnaire is 94.4% and the specificity is 14.3%. Therefore, endoscopy is useful in lowering the number of false positives. However, as the goal of treatment is the control of symptoms, the value of nasal endoscopy in defining control is one that is debatable.

